# Supplementary figures and images for: Callus growth kinetics and accumulation of secondary metabolites of Bletilla striata Rchb.f. using a callus suspension culture
Source: PLoS One. 2020 Feb 19;15(2):e0220084. doi: 10.1371/journal.pone.0220084 (PMC7029869; doi:10.1371/journal.pone.0220084)

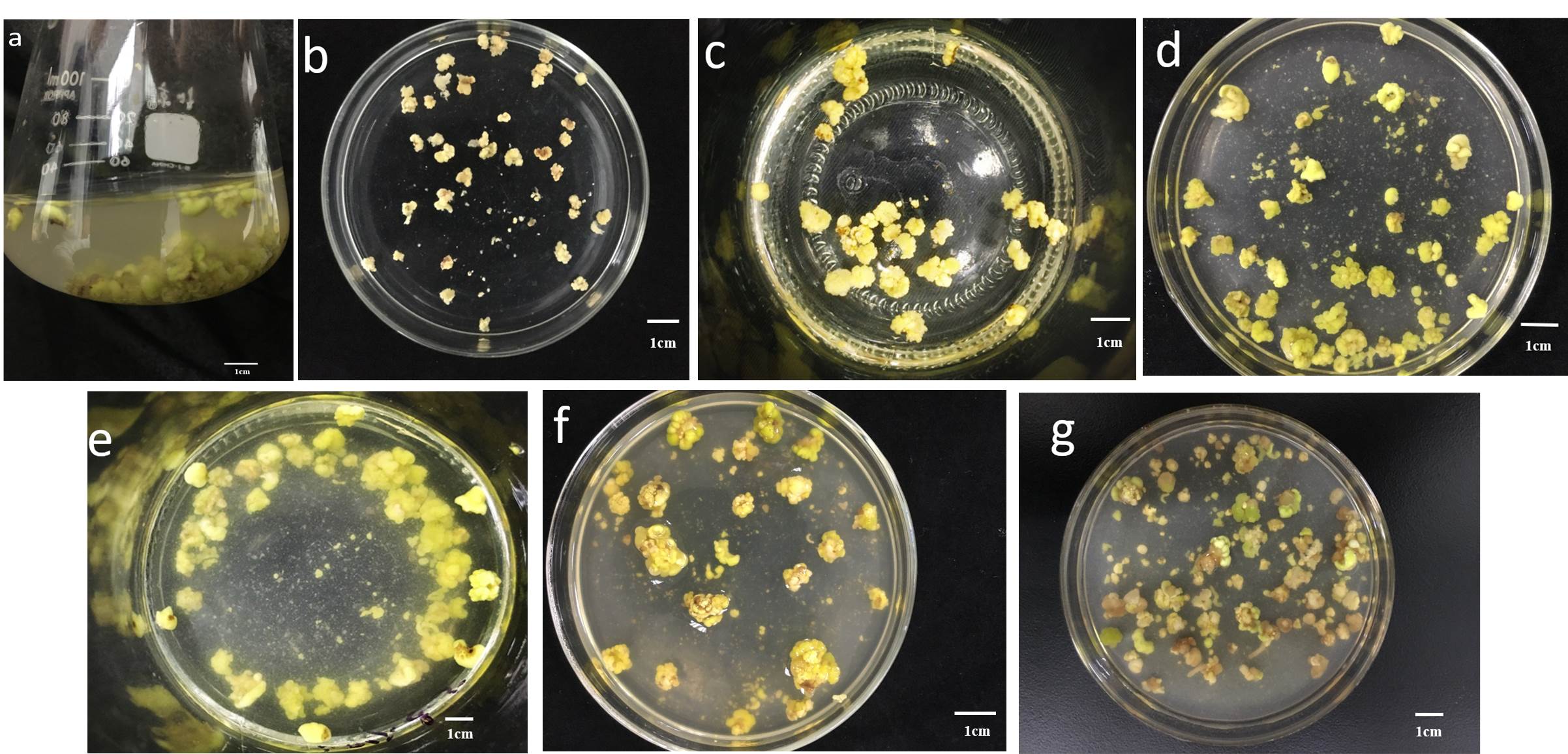

Supplement: S1 Fig — a. Suspension culture system. b. Calluses collected from the culture system after 3 days of incubation. c. Calluses after 9 days of incubation. d. Calluses collected from the culture system after 18 days of incubation. e. Calluses after 27 days of incubation. f. Calluses collected from the culture system after 33 days of incubation. g. Calluses collected from the culture system after 45 days of incubation. (JPG) [file pone.0220084.s001.jpg]

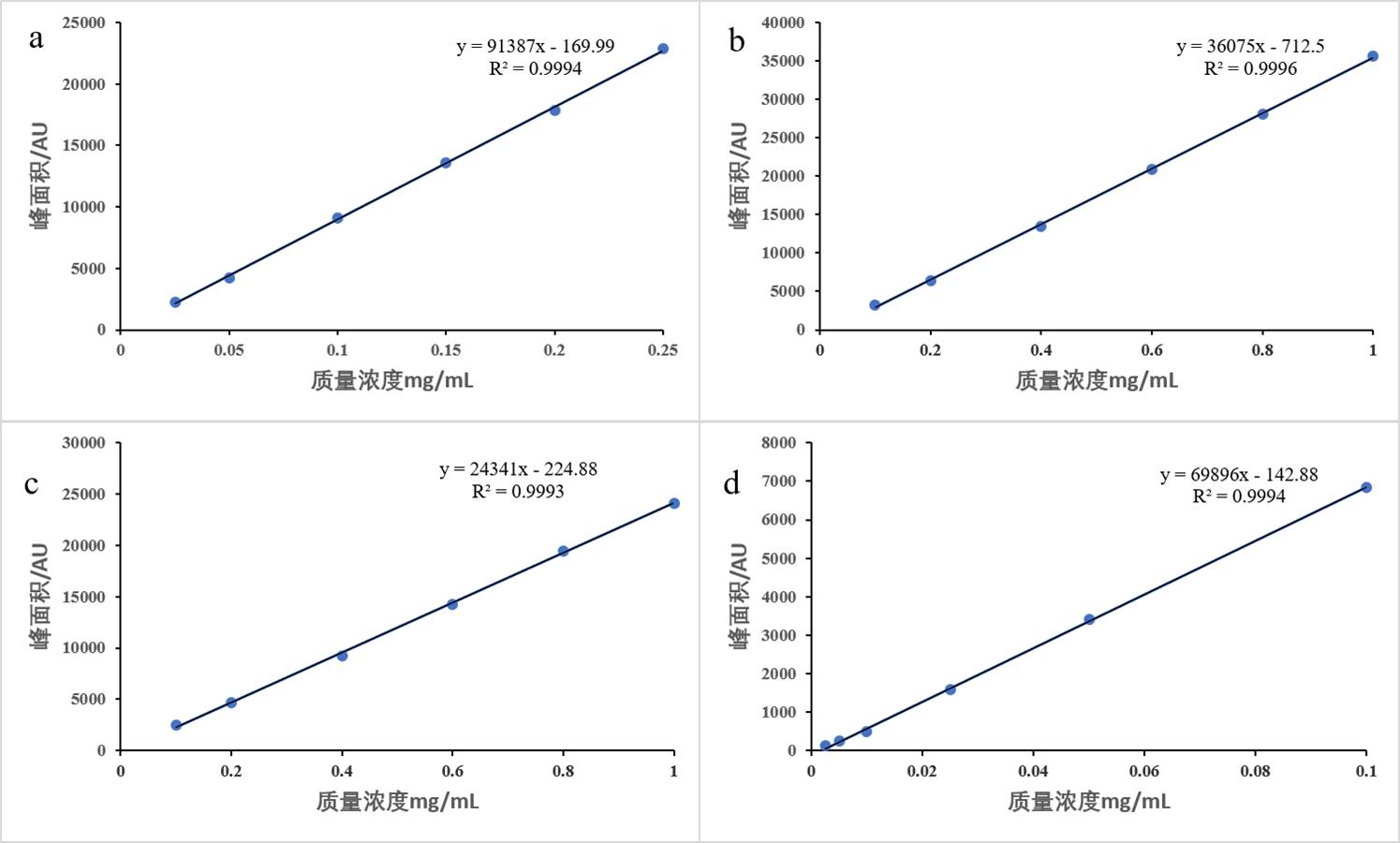

Supplement: S2 Fig — a. 4-Hydroxybenzyl alcohol. b. Dactylorhin A. c. Militarine; d. Coelonin. (JPG) [file pone.0220084.s002.jpg]
