# Supplementary material for: Callus growth kinetics and accumulation of secondary metabolites of Bletilla striata Rchb.f. using a callus suspension culture
Source: PLoS One. 2020 Feb 19;15(2):e0220084. doi: 10.1371/journal.pone.0220084 (PMC7029869; doi:10.1371/journal.pone.0220084)
Supplement: S1 Table — (DOCX) [file pone.0220084.s003.docx]

Table S1. Dry and wet weight of suspension cultured callus in 45-day culture period.

| days | Average dry weight | Dry weight standard error | Average fresh weight | Fresh weight standard error |
| --- | --- | --- | --- | --- |
| 0 | 0.1032 | 0.0015 | 1.054 | 0.0568 |
| 3 | 0.1251 | 0.0057 | 1.2213 | 0.0119 |
| 6 | 0.1414 | 0.0021 | 1.3779 | 0.0121 |
| 9 | 0.1681 | 0.0088 | 1.5038 | 0.0451 |
| 12 | 0.2265 | 0.0091 | 2.0726 | 0.1128 |
| 15 | 0.2838 | 0.0045 | 2.5044 | 0.0317 |
| 18 | 0.3153 | 0.0139 | 3.0615 | 0.2026 |
| 21 | 0.3352 | 0.0058 | 3.3761 | 0.0675 |
| 24 | 0.3678 | 0.0103 | 3.5119 | 0.037 |
| 27 | 0.3476 | 0.0063 | 3.4317 | 0.2091 |
| 30 | 0.3679 | 0.0088 | 4.334 | 0.1297 |
| 33 | 0.3855 | 0.0049 | 4.4423 | 0.2473 |
| 36 | 0.4292 | 0.019 | 4.729 | 0.0859 |
| 39 | 0.4196 | 0.0176 | 4.6067 | 0.0187 |
| 42 | 0.3962 | 0.0087 | 4.5252 | 0.044 |
| 45 | 0.3809 | 0.0073 | 4.4479 | 0.0931 |
