# Supplementary material for: Callus growth kinetics and accumulation of secondary metabolites of Bletilla striata Rchb.f. using a callus suspension culture
Source: PLoS One. 2020 Feb 19;15(2):e0220084. doi: 10.1371/journal.pone.0220084 (PMC7029869; doi:10.1371/journal.pone.0220084)
Supplement: S2 Table — (DOCX) [file pone.0220084.s004.docx]

Table S2 Precision examination results of HPLC detection

| Secondary metabolites |  | 1 | 2 | | 3 | | 4 | | 5 | | RSD(%) | |
| --- | --- | --- | --- | --- | --- | --- | --- | --- | --- | --- | --- | --- |
| 4-hydroxybenzyl alcohol | Retention time (min) | 10.660 | | 10.651 | | 10.649 | | 10.653 | | 10.634 | | 0.09 |
|  | Peak area (mAU) | 4189.6 | | 4190.0 | | 4188.0 | | 4269.1 | | 4219.9 | | 0.83 |
| dactylorhin A | Retention time (min) | 31.262 | | 31.243 | | 31.251 | | 31.225 | | 31.292 | | 0.08 |
|  | Peak area (mAU) | 13482.0 | | 13563.1 | | 13530.8 | | 13418.4 | | 13492.4 | | 0.40 |
| militarine | Retention time (min) | 36.074 | | 36.105 | | 36.046 | | 36.042 | | 36.074 | | 0.07 |
|  | Peak area (mAU) | 14223.8 | | 14295.1 | | 14705.1 | | 14305.7 | | 14275.4 | | 1.36 |
| coelonin | Retention time (min) | 40.529 | | 40.532 | | 40.564 | | 40.532 | | 40.680 | | 0.16 |
|  | Peak area (mAU) | 459.6 | | 464.8 | | 463.8 | | 471.9 | | 456.9 | | 1.24 |
