# Supplementary material for: Callus growth kinetics and accumulation of secondary metabolites of Bletilla striata Rchb.f. using a callus suspension culture
Source: PLoS One. 2020 Feb 19;15(2):e0220084. doi: 10.1371/journal.pone.0220084 (PMC7029869; doi:10.1371/journal.pone.0220084)
Supplement: S3 Table — (DOCX) [file pone.0220084.s005.docx]

Table S3 Stability test results of HPLC detection

| Secondary metabolites |  | 0 | 3 | 6 | 9 | 12 | RSD(%) |
| --- | --- | --- | --- | --- | --- | --- | --- |
| 4-hydroxybenzyl alcohol | Retention time (min) | 10.809 | 10.713 | 10.916 | 10.928 | 10.915 | 0.86 |
|  | Peak area (mAU) | 4572.5 | 4569.5 | 4595.4 | 4507.8 | 4555.6 | 0.71 |
| dactylorhin A | Retention time (min) | 31.378 | 31.324 | 31.609 | 31.655 | 31.648 | 0.50 |
|  | Peak area (mAU) | 34422.1 | 34042.8 | 35027.4 | 35059.6 | 35102.2 | 1.37 |
| militarine | Retention time (min) | 36.209 | 36.122 | 36.629 | 36.714 | 36.656 | 0.76 |
|  | Peak area (mAU) | 13124.7 | 12868.2 | 12539.1 | 12770.8 | 12579.5 | 1.86 |
| coelonin | Retention time (min) | 40.960 | 40.920 | 41.399 | 40.020 | 39.882 | 1.61 |
|  | Peak area (mAU) | 1431.0 | 1456.6 | 1511.2 | 1418.7 | 1411.2 | 2.80 |
