# Supplementary material for: Callus growth kinetics and accumulation of secondary metabolites of Bletilla striata Rchb.f. using a callus suspension culture
Source: PLoS One. 2020 Feb 19;15(2):e0220084. doi: 10.1371/journal.pone.0220084 (PMC7029869; doi:10.1371/journal.pone.0220084)
Supplement: S4 Table — (DOCX) [file pone.0220084.s006.docx]

Table S4 Repeatability test results of HPLC detection

| Secondary metabolites |  | 1 | 2 | 3 | 4 | 5 | RSD(%) |
| --- | --- | --- | --- | --- | --- | --- | --- |
| 4-hydroxybenzyl alcohol | Retention time (min) | 10.800 | 10.843 | 10.867 | 10.734 | 10.764 | 0.51 |
|  | Peak area (mAU) | 4162.7 | 3997.1 | 4004.4 | 3959.5 | 3972.6 | 2.05 |
|  | Contents（mg/g） | 1.19 | 1.14 | 1.14 | 1.13 | 1.13 | 2.19 |
| dactylorhin A | Retention time (min) | 31.391 | 31.408 | 31.419 | 31.336 | 31.341 | 0.12 |
|  | Peak area (mAU) | 32283.7 | 34390.6 | 33235.8 | 33508.8 | 32419.0 | 2.59 |
|  | Contents（mg/g） | 22.87 | 24.33 | 23.53 | 23.72 | 22.96 | 2.54 |
| militarine | Retention time (min) | 36.259 | 36.313 | 36.262 | 36.227 | 36.108 | 0.21 |
|  | Peak area (mAU) | 13005.1 | 13136.3 | 12994.1 | 12858.5 | 13088.2 | 0.82 |
|  | Contents（mg/g） | 13.59 | 13.72 | 13.58 | 13.44 | 13.67 | 0.78 |
| coelonin | Retention time (min) | 41.048 | 41.288 | 41.084 | 41.594 | 41.013 | 0.59 |
|  | Peak area (mAU) | 1388.8 | 1330.9 | 1407.6 | 1422.8 | 1389.7 | 2.51 |
|  | Contents（mg/g） | 0.55 | 0.53 | 0.55 | 0.56 | 0.55 | 2.00 |
