# Supplementary material for: Callus growth kinetics and accumulation of secondary metabolites of Bletilla striata Rchb.f. using a callus suspension culture
Source: PLoS One. 2020 Feb 19;15(2):e0220084. doi: 10.1371/journal.pone.0220084 (PMC7029869; doi:10.1371/journal.pone.0220084)
Supplement: S5 Table — (DOCX) [file pone.0220084.s007.docx]

Table S5 Recovery test results of tested chemicals

| Secondary metabolites | Original quantity（mg） | Amount added（mg） | Measured amount（mg） | Recovery rate（%） | The average recovery rate（%） | RSD(%) |
| --- | --- | --- | --- | --- | --- | --- |
| 4-hydroxybenzyl | 0.0281 | 0.0300 | 0.0575 | 99.04 | 99.15  98.32  96.98  99.59 | 1.59  1.85  1.24  1.98 |
| alcohol | 0.0244 | 0.0300 | 0.0539 | 99.08 |  |  |
|  | 0.0241 | 0.0300 | 0.0545 | 100.74 |  |  |
|  | 0.0233 | 0.0250 | 0.0481 | 99.59 |  |  |
|  | 0.0242 | 0.0250 | 0.0493 | 100.20 |  |  |
| dactylorhin A  militarine  coelonin | 0.0261  0.0249  0.0255  0.0231  0.5612  0.4876  0.4807  0.4646  0.4830  0.5221  0.4968  0.5083  0.4623  0.3318  0.2883  0.2842  0.2747  0.2855  0.3087  0.2937  0.3005  0.2733  0.0137  0.0119  0.0117  0.0113  0.0118  0.0127  0.0121  0.0124  0.0113 | 0.0250  0.0200  0.0200  0.0200  0.6000  0.6000  0.6000  0.5000  0.5000  0.5000  0.4000  0.4000  0.4000  0.3500  0.3500  0.3500  0.2900  0.2900  0.2900  0.2320  0.2320  0.2320  0.0150  0.0150  0.0150  0.0125  0.0125  0.0125  0.0100  0.0100  0.0100 | 0.0492  0.0447  0.0459  0.0418  1.1181  1.0530  1.0561  0.9627  0.9547  1.0333  0.8894  0.9164  0.8317  0.6708  0.6178  0.6233  0.5533  0.5501  0.5824  0.5041  0.5193  0.4807  0.0284  0.0278  0.0269  0.0232  0.0238  0.0249  0.0218  0.0220  0.0217 | 96.28  99.55  100.88  96.98  96.29  96.82  97.72  99.80  97.12  100.64  99.17  100.89  96.45  98.39  96.79  98.28  97.98  95.59  97.28  95.89  97.48  95.13  98.95  103.35  100.75  97.48  97.94  98.81  98.94  98.21  101.88 |  |  |
