# Supplementary material for: Callus growth kinetics and accumulation of secondary metabolites of Bletilla striata Rchb.f. using a callus suspension culture
Source: PLoS One. 2020 Feb 19;15(2):e0220084. doi: 10.1371/journal.pone.0220084 (PMC7029869; doi:10.1371/journal.pone.0220084)
Supplement: S6 Table — (DOCX) [file pone.0220084.s008.docx]

Table S6. Mean measurement of secondary metabolites in 45-day culture period.

| days | p-hydroxybenzyl | dactylorhin A | militarine | coelonin |
| --- | --- | --- | --- | --- |
| 0 | 0.3171 | 6.7489 | 2.2007 | 0.1658 |
| 3 | 0.4512 | 8.0013 | 2.9262 | 0.1851 |
| 6 | 0.5275 | 8.4768 | 2.9545 | 0.1986 |
| 9 | 0.6489 | 9.0743 | 2.9832 | 0.2625 |
| 12 | 0.6719 | 10.316 | 3.3472 | 0.2674 |
| 15 | 0.6774 | 10.329 | 3.5668 | 0.2954 |
| 18 | 0.7025 | 11.4963 | 4.8781 | 0.3323 |
| 21 | 0.7168 | 13.4529 | 5.452 | 0.315 |
| 24 | 0.82 | 19.0204 | 6.3178 | 0.2788 |
| 27 | 0.9114 | 13.4212 | 5.1489 | 0.2649 |
| 30 | 0.9368 | 12.2209 | 4.2688 | 0.24 |
| 33 | 1.0178 | 11.0534 | 3.9618 | 0.2243 |
| 36 | 1.0327 | 8.569 | 3.0688 | 0.2157 |
| 39 | 1.0489 | 8.0872 | 2.8821 | 0.2116 |
| 42 | 1.0221 | 6.7064 | 2.5375 | 0.2094 |
| 45 | 0.9865 | 6.7056 | 2.3086 | 0.201 |
