# Supplementary material for: Callus growth kinetics and accumulation of secondary metabolites of Bletilla striata Rchb.f. using a callus suspension culture
Source: PLoS One. 2020 Feb 19;15(2):e0220084. doi: 10.1371/journal.pone.0220084 (PMC7029869; doi:10.1371/journal.pone.0220084)
Supplement: Attachment — Submitted filename: Response to Reviewers.docx [file pone.0220084.s009.docx]

**Response to Reviewers**

**Dear Editor and Reviewers:**

Thanks for your letter and the comments on our manuscript “Cell growth kinetics and accumulation of secondary metabolite of *Bletilla striata* Rchb.f. using cell suspension culture”.

We have learned much from the comments, which are fair, encouraging and also constructive. After careful consideration, corresponding addresses were made to our submitted manuscript. And the reviewer’s comments were responded point-point as fellows, which along with a clear indication of the location of the revised version. Hope these changes will make it fully meet PLOS ONE’s publication criteria. The main comments and our specific responses are detailed as below:

Reviewer #1: In this study, the authors cultivate suspension culture of *Bletilla striata* callus cells and recorded the changes in fresh weight, dry weight and secondary metabolite content during culture, which has certain significance for the sustainable use of Bletilla striata resources. However, I suggest that this manuscript needs to undergo major revision.

1. Please add figures of *Bletilla striata* callus at different stages.

Response: The figures of different stages of *B. striata* callus were added in Figure S1 (stated in Line 202).

2. In the abstract, "*Bletilla striata* is a well-recognized endangered medicinal plant due to inadequate natural reproduction with high market worth. "This statement is exaggerated, please describe it in a suitable vocabulary.

Response: According to your suggestion, we have revised this statement as “*Bletilla striata* is an endangered traditional Chinese medicinal plant with multiple uses and slow pace on regeneration of germplasm resource” in line 22 and 23 of the Revised manuscript.

3. Does the dry weight of the sample affect the number of subsequent experimental samples?

Response: As we explained in the article, the material used in the experiment was a uniform callus derived from *B. striata* seeds of the same genotype. Therefore, in the same batch of materials, although the number of subsequent experimental samples is limited due to the dry weight of the sample. However, the subsequent experimental materials can be repeated according to our experimental methods, and the same experimental results can be obtained.

4. English needs to be completely reviewed by a native speaker.

Response: According to your suggestion, this manuscript has been modified by a proferssor whose native language is English. And the English polishing tracks could be found in attached file of “Revised Manuscript with Track Changes”, which was edited by American Journal Experts (AJE) company with the supporting file of “Certification by AJE”.

Reviewer #2:

1. The work is well done and presented. However, the material generated by the Authors did not respected the full description for a cell suspension (homogeneity or cellular individualization, for example), because this research with the initial steps of cellular individualization. It could be worth to mention this point.

Response: All the inoculated materials were derived from the uniform callus induced by the seeds in the mature capsules of the same artificial sister on the same single plant in Zheng’an County, Guizhou Province, China(28°56′N, 107°43′E). According to your suggestion, we have added this information in our Methods section of the Revised manuscript from Line 92 to Line 94.

2. p-hydroxybenzyl alcohol: p in italic. This work is in my opinion could be acceptable for publication following these changes.

Response: The spelling mistake of *p*-hydroxybenzyl alcohol has been corrected in Line 41, 84, 111, 161, 164, 174, 180, 257, 262, 266, 288, 307, 331, 343, 345, 347 and 476.
